# Enhancement of low‐fat Feta cheese characteristics using probiotic bacteria

**DOI:** 10.1002/fsn3.1889

**Published:** 2020-11-26

**Authors:** Ahmed M. Hamdy, Mahmoud E. Ahmed, Dipakkumar Mehta, Mohamed Salem Elfaruk, Ahmed R. A. Hammam, Yaser M. A. El‐Derwy

**Affiliations:** ^1^ Dairy Science Department Faculty of Agriculture Assiut University Assiut Egypt; ^2^ Research and Development Wells Enterprises Inc. Le Mars IA USA; ^3^ Dairy and Food Science Department South Dakota State University Brookings SD USA; ^4^ Medical Technology College Nalut University Nalut Libya

**Keywords:** cheese microbiology, feta cheese, flavor, low‐fat, probiotic adjuncts, sensory analysis

## Abstract

The objective of this study was to manufacture low‐fat Feta cheese (LFC) using different types of starter cultures, such as yogurt (Y) cultures (*Streptococcus thermophilus* and *Lactobacillus bulgaricus*), bifidobacterium (B) cultures (*Bifidobacterium bifidum* and *Bifidobacterium longum*), and mixed of them (Y + B) at different rates (0.4, 0.5, and 0.6%). The Y + B cultures improved the flavor and body and texture of LFC, especially at a ratio of 0.4 + 0.6% and 0.5 + 0.5%, which is similar to the typical full‐fat Feta cheese. Also, the LFC maintained a higher number of probiotics and lactic acid bacteria after 30 d of storage at a range of 5 to 7 log cfu/g.

## Graphical abstract

The objective of this study was to manufacture low‐fat Feta cheese (LFC) using different types of starter cultures, such as yogurt (Y) cultures (Streptococcus thermophilus and Lactobacillus bulgaricus), bifidobacterium (B) cultures (Bifidobacterium bifidum and Bifidobacterium longum), and mixed of them (Y+B) at a different rate (0.4, 0.5, and 0.6%). The Y+B cultures improved the flavor and body and texture of LFC, especially at a ratio of 0.4+0.6% and 0.5+0.5%, which is similar to the typical full‐fat Feta cheese (FC). Also, the LFC maintained a higher number of probiotics and lactic acid bacteria after 30 d of storage.

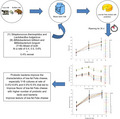

## 1 INTRODUCTION

Feta cheese is a brined soft cheese and it is widely popular in many countries in Africa, Europe, and other regions. It was manufactured from goat milk, but nowadays different types of milk, including sheep, cow, and buffalo milk are utilized to produce this type of cheese. Traditionally, Feta cheese was made from raw milk to decrease the ripening period, as well as, having the intense flavor that results from the natural microflora. However, milk is pasteurized nowadays before being used in making Feta cheese to assure the safety of consumers and to maintain the typical characteristics of cheese. Manufacturing of Feta cheese from pasteurized milk has various challenges due to the lack of flavor intensity as compared to Feta cheese made from raw milk (Bintsis & Robinson, 2004).

Consequently, several studies have focused on producing Feta cheese from pasteurized milk using different types of starter cultures, additives, various sources of milk and different processing conditions to stimulate the flavor and texture of the raw Feta cheese. Feta cheese is typically manufactured using lactic acid bacteria (LAB). The count of LAB in Feta cheese is increasing with elevating the acidity at the beginning of the ripening at 5–7°C, and then the count of LAB becomes constant up to 60 d (Bintsis & Robinson, 2004). On the other hand, the count of mesophilic starter cultures is decreasing at the prematuration of Feta cheese, especially at a higher salt content (6%–8%) and pH of < 5.0. As a result, thermophilic and probiotic bacteria have been used as adjunct starter cultures to enhance the flavor of Feta cheese. The microflora in cheese produces the typical flavor by their metabolic activities, such as proteolysis, lipolysis, and lactose fermentation. Several probiotic cultures, such as bifidobacterium and streptococci (Peirotén, Gaya, Arqués, Medina, & Rodríguez, 2019), have been utilized recently in the manufacture of cheese due to their ability to produce the desirable flavors (Champagne, Gagnon, St‐Gelais, & Vuillemard, 2009). Furthermore, probiotic bacteria can provide health benefits to the consumers at the rate of 5–7 log cfu/g present in the product (Roy, 2001).

Full‐fat Feta cheese (FFC) can result in several health issues, such as high blood pressure, cardiovascular diseases, increased cholesterol levels, and obesity due to the high fat content. The low‐fat Feta cheese (LFC) is healthy and beneficial for dieters and heart patients, especially when it is manufactured with probiotics that have several promising health benefits. Although the production of LFC has many challenges due to the lack of fat which in turn has a significant effect on the flavor and texture characteristics of cheese, the sales and market growth of LFC have increased recently due to the negative effect of FFC on health (Michaelidou, Katsiari, Kondyli, Voutsinas, & Alichanidis, 2003). As a result, many researchers have been working on manufacturing of LFC by mimicking its flavor and texture characteristics to that of the FFC (Bintsis & Robinson, 2004). Several modifications have been applied to the traditional process of making LFC to compensate for the lack of fat, such as using different starter cultures and increasing the moisture content of cheese to make the texture smoother. The most common and efficient method, is using probiotic bacteria as adjunct cultures to improve the flavor and texture of cheese as compared to FFC. Those bacteria have a higher metabolism which can produce sufficient flavor components, resulting in a typical FFC flavor. The probiotic adjunct cultures have well improved LFC properties as compared to the conventional starter cultures that are typically used to manufacture Feta cheese. Several studies have been performed to improve the quality of LFC, which is made from partially skimmed bovine milk. To date, there is no study that describes improving the characteristics of LFC from mixed skim milk (cow and buffalo milk) by using different types of starter cultures at different ratios. Therefore, the objective of this study was to manufacture LFC from mixed skim milk (cow and buffalo milk) using yogurt cultures (Y) (*Streptococcus thermophilus* and *Lactobacillus bulgaricus*), Bifidobacterium cultures (B) (*Bifidobacterium bifidum* and *Bifidobacterium longum*), and mixed of them (Y + B) at different rates (0.4, 0.5, and 0.6%) and evaluate the microbiological and sensory characteristics during 30 d of storage.

## 2 MATERIAL AND METHODS

### 2.1 Manufacturing of low‐fat Feta cheese with probiotics

The LFC was manufactured from a mix of cow and buffalo skim milk. Fresh milk was obtained from the Animal Production Farm (Faculty of Agriculture, Assiut University, Assiut, Egypt) and was separated at 20°C. Cow skim milk and buffalo skim milk were mixed at a ratio of 1:1, heated at 73°C/16 s, and then cooled to 40°C. The skim milk was divided into sixteen lots, and then 3% of sodium chloride was added to each part. Then, the starter cultures were added for each lot as shown in Table 1. The Y and B cultures were obtained from Cairo Microbiological Resources Center, Faculty of Agriculture, Ain Shams University, Cairo, Egypt. The first part of skim milk (control) was coagulated by adding 0.4% of rennet (Chr. Hansen, Copenhagen, Denmark), while the other fifteen lots of skim milk were turned into cheese by using rennet plus Y cultures at a rate of 0.4% (T1), 0.5% (T2), and 0.6% (T3), B cultures at a percentage of 0.4% (T4), 0.5% (T5), and 0.6% (T6), and mixture of Y + B at a percentage of 0.4 + 0.4% (T7), 0.4 + 0.5% (T8), 0.4 + 0.6% (T9), 0.5 + 0.4% (T10), 0.5 + 0.5% (T11), 0.5 + 0.6% (T12), 0.6 + 0.4% (T13), 0.6 + 0.5% (T14), 0.6 + 0.6% (T15). A 0.4% of rennet was added to other treatments after 1 hr of starter culture addition.

**TABLE 1:** The starter cultures and percentage used to manufacture low‐fat Feta cheese (LFC)

Starter cultures	Treatment	Rennet (%)	Starter cultures (%)
None	Control		None
Group (Y) *Streptococcus thermophilus* and *Lactobacillus bulgaricus* (1:1)	T1		0.4
	T2		0.5
	T3		0.6
Group (B) *Bifidobacterium bifidum* and *Bifidobacterium longum* (1:1)	T4		0.4
	T5		0.5
	T6		0.6
Group (Y + B)	T7	0.4	0.4 + 0.4
	T8		0.4 + 0.5
	T9		0.4 + 0.6
	T10		0.5 + 0.4
	T11		0.5 + 0.5
	T12		0.5 + 0.6
	T13		0.6 + 0.4
	T14		0.6 + 0.5
	T15		0.6 + 0.6

The inoculated skim milk was left for 30 min at 40°C until complete coagulation. Then, the curd was cut, packed in cheesecloth, and left for draining overnight at 5°C. The cheese was removed from the cheesecloth on the following day, cut into cubes, and pickled in sterilized glass containers contained whey that produced during making the cheese. Cheeses from different treatments were stored at 6 ± 2°C and analyzed when fresh (d = 0), and after 7, 15, 21, and 30 d. This experiment was repeated three times.

### 2.2 Microbiological analyses

One gram of the cheese samples was weighed under an aseptic environment and transferred into a sterilized jar. Subsequently, 9 ml of a sterile phosphate buffer was added and evenly mixed to have the 1:10 dilution, which was further used to prepare the sequence of dilutions (Mehta, Metzger, Hassan, Nelson, & Patel, 2019). Total bacterial count (TBC) was enumerated by using the standard plate count technique (Wehr & Frank, 2004). The proper dilutions of the samples were plated in duplicates on a nutrient agar medium. The plated medium was inoculated at 32°C for 48–72 hr before the colonies being enumerated. Lactobacilli counts (LC) were determined using the MRS agar medium according to De Man, Rogosa, and Sharpe (1960). All plates were anaerobically incubated at 37°C for 48 hr. Streptococci count (SC) was enumerated using the M17 agar medium (Wehr & Frank, 2004). The plates were anaerobically incubated at 40°C for 48 hr. Bifidobacterium count (BC) was enumerated using a fluid thioglycollate medium using a pouring plat method (Brewer, 1940). The plates were anaerobically incubated at 37°C for 48 hr. The coliform count was enumerated on MacConkey broth media, and tubes were incubated at 32°C ± 1°C for 24 hr (Ashenafi, 1990). Yeast and mold count were also enumerated (Wehr & Frank, 2004) using potato dextrose agar media, and plates were incubated at 25°C ± 1°C for 5 d. The microbiological analyses were performed at 0, 7, 15, 21, and 30 d.

### 2.3 Sensory evaluation

The sensory characteristics of LFC samples were evaluated according to 10–15 trained panelists from the Dairy Science Department, Assiut University. The LFC was examined as described by Mehta with some modifications (Mehta, Kumar, & Sabikhi, 2017). Samples were evaluated for color and appearance (15 points), flavor (50 points), and body and texture (35 points) to have 100 points overall. The organoleptic characteristics were evaluated at 0, 15, and 30 d of storage.

### 2.4 Statistical analysis

Results were analyzed by R software (R x64‐3.3.3, 9,205 NW 101st St, Miami, Florida, United States). All data were analyzed by ANOVA using a GLM for each variable to study the effect of probiotic bacteria and time on the characteristics of LFC. Mean separation was done using the least significant difference (LSD) comparison test when significant differences were detected at *p* < .05.

## 3 RESULTS AND DISCUSSION

### 3.1 Microbiological analyses

**FIGURE 1:**
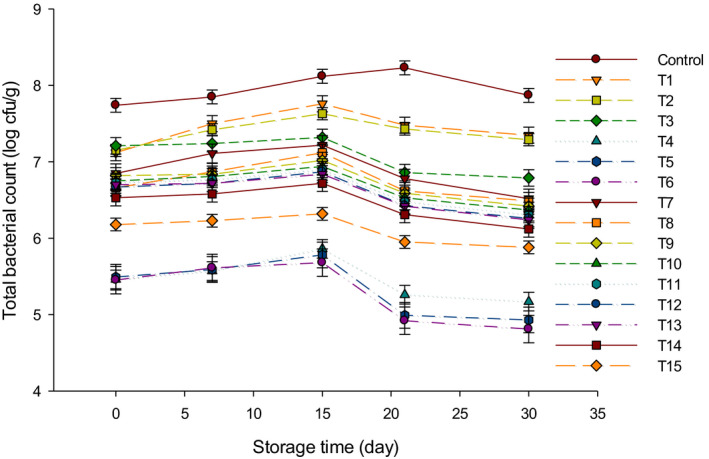
The total bacterial counts (log cfu/g) of low‐fat Feta cheese (LFC) during 30 d of storage period. Control = Cheese made with only rennet; Group (Y) = 0.4% (T1), 0.5% (T2), and 0.6% (T3); Group (B) = 0.4% (T4), 0.5% (T5), and 0.6% (T6); Group (Y + B) = 0.4Y + 0.4B (T7), 0.4Y + 0.5B (T8), 0.4Y + 0.6B (T9), 0.5Y + 0.4B (T10), 0.5Y + 0.5B (T11), 0.5Y + 0.6B (T12), 0.6Y + 0.4B (T13), 0.6Y + 0.5B (T14), 0.6Y + 0.6B (T15). Yogurt cultures (Y) (*Streptococcus thermophilus* and *Lactobacillus bulgaricus*) and bifidobacterium cultures (B) (*Bifidobacterium bifidum* and *Bifidobacterium longum*)

Figure 1 is exemplified the TBC of LFC samples through 30 d of storage. The TBC increased during the first 15 d of storage in all treatments except in control which increased up to 21 d, subsequently, it decreased until the 30 d of storage. The TBC of control cheese was higher as compared to other treatments. This could be due to the antimicrobial activity of probiotic bacteria or the high acidity content (Ahmed, Hamdy, El‐Derway, El‐Gazzar, & El‐Naga, 2020) that may have limited the growth of bacteria in other treatments (Charlier, Even, Gautier, & Le Loir, 2008). Cheeses of group Y (T1, T2, and T3) had higher TBC as compared to other groups. By the end of ripening, the highest TBC was 7.87 log cfu/g in control while the lowest was 4.81 log cfu/g for T6 as compared to other treatments. The TBC decreased with the increase of starter cultures, and this could be caused by acidity development (Ahmed et al., 2020), which led to the inhibition of bacteria in LFC (Charlier et al., 2008). The TBC in group B cheese (T4, T5, and T6) was the lowest as compared to other groups, and this can be related to the medium which is not suitable as a selective media for bifidobacterium or the poor growth of bifidobacteria (Peirotén et al., 2019). On the other hand, mixing groups Y and B did not markedly increase (*p* > .05) the TBC. This may be due to the high acidity levels in this group during the storage period which limits the growth of microorganisms (Ahmed et al., 2020). These results were in agreement with other studies that reported the TBC in soft cheese made with probiotic increased up to 15 d of storage, and then, the number decreased until the end of storage (Hassanien, Mahgoub, & El‐Zahar, 2014).

**FIGURE 2:**
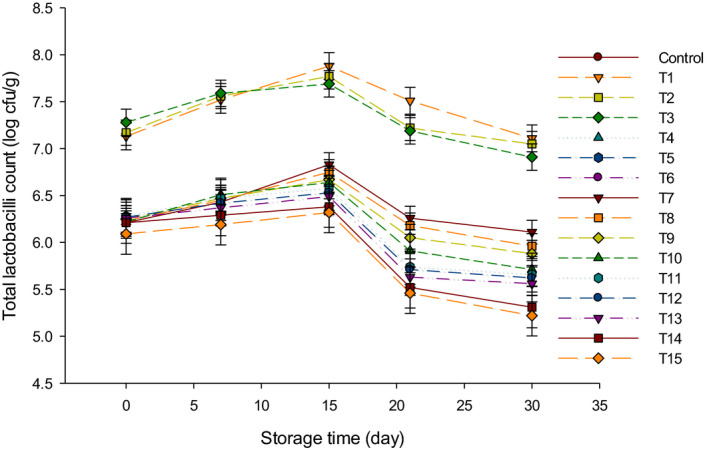
The total lactobacilli counts (log cfu/g) of low‐fat Feta cheese (LFC) during 30 d of storage period. Control = Cheese made with only rennet; Group (Y) = 0.4% (T1), 0.5% (T2), and 0.6% (T3); Group (B) = 0.4% (T4), 0.5% (T5), and 0.6% (T6); Group (Y + B) = 0.4Y + 0.4B (T7), 0.4Y + 0.5B (T8), 0.4Y + 0.6B (T9), 0.5Y + 0.4B (T10), 0.5Y + 0.5B (T11), 0.5Y + 0.6B (T12), 0.6Y + 0.4B (T13), 0.6Y + 0.5B (T14), 0.6Y + 0.6B (T15). Yogurt cultures (Y) (*Streptococcus thermophilus* and *Lactobacillus bulgaricus*) and bifidobacterium cultures (B) (*Bifidobacterium bifidum* and *Bifidobacterium longum*)

Figure 2 presented the LC in LFC during 30 d of storage. The LC was not detected in control and group B cheeses because lactobacilli cultures were not added during manufacturing of those cheeses. Group Y recorded the highest numbers of lactobacilli during 30 d of storage. The highest number of lactobacilli was 7.88 log cfu/g in T1 after 15 d, and it decreased to 7.11 log cfu/g after 30 d, while the lowest LC number was observed in T15 and reached 5.22 log cfu/g after 30 d. The LC followed the same trend as TBC and decreased after 15 d of storage. This could be due to the increase in acidity (Ahmed et al., 2020) that led to inhibition of LC in cheese (Charlier et al., 2008). For the same reason, the LC in cheeses made form group Y + B was the lowest during 30 d of storage. It has been reported that lactobacilli had low resistance (Hammam & Ahmed, 2019) under the extended acidic condition which is similar to our study.

**FIGURE 3:**
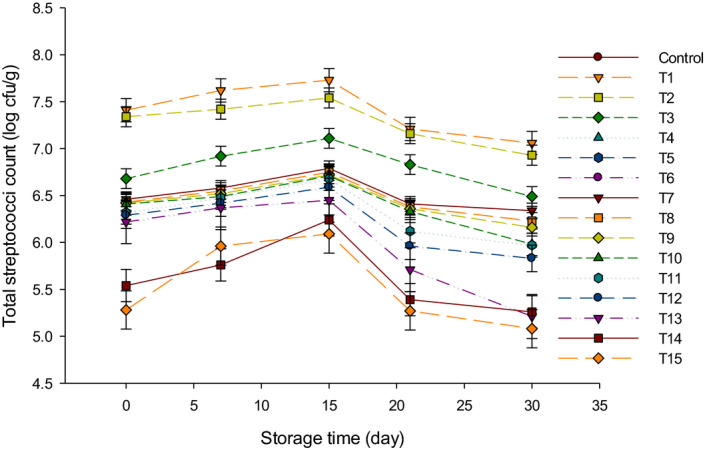
The total streptococci counts (log cfu/g) of low‐fat Feta cheese (LFC) during 30 d of storage period. Control = Cheese made with only rennet; Group (Y) = 0.4% (T1), 0.5% (T2), and 0.6% (T3); Group (B) = 0.4% (T4), 0.5% (T5), and 0.6% (T6); Group (Y + B) = 0.4Y + 0.4B (T7), 0.4Y + 0.5B (T8), 0.4Y + 0.6B (T9), 0.5Y + 0.4B (T10), 0.5Y + 0.5B (T11), 0.5Y + 0.6B (T12), 0.6Y + 0.4B (T13), 0.6Y + 0.5B (T14), 0.6Y + 0.6B (T15). Yogurt cultures (Y) (*Streptococcus thermophilus* and *Lactobacillus bulgaricus*) and bifidobacterium cultures (B) (*Bifidobacterium bifidum* and *Bifidobacterium longum*)

The results of total SC are illustrated in Figure 3. No SC colonies were found either in control or group B cheeses because those cheeses were not manufactured using streptococci cultures. Group Y recorded the highest numbers of SC during 30 d of storage as compared to group Y + B. After 15 d of storage, the highest SC was found in T1 with 7.73 log cfu/g while the lowest SC was detected in T15 with 6.09 log cfu/g. The total SC increased up to 15 d of storage and decreased until the end of storage, which was similar to TBC and LC trends. These results were in agreement with another study that found *Streptococcus thermophilus* count increased up to 15 d of storage in soft white cheese, and then, it decreased after 28 d (Yerlikaya & Ozer, 2014). Group Y + B contained the lowest numbers of SC in LFC during 30 d of storage as compared with other groups due to the higher acidity content in this group as mentioned in our previous study (Ahmed et al., 2020).

**FIGURE 4:**
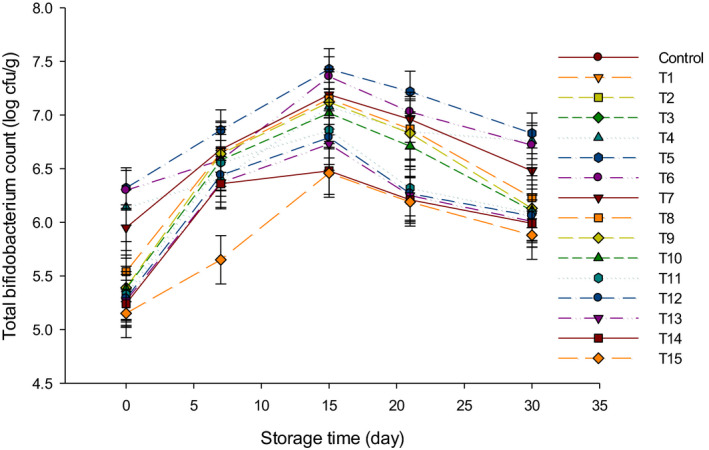
The total Bifidobacterium counts (log cfu/g) of low‐fat Feta cheese (LFC) during 30 d of storage period. Control = Cheese made with only rennet; Group (Y) = 0.4% (T1), 0.5% (T2), and 0.6% (T3); Group (B) = 0.4% (T4), 0.5% (T5), and 0.6% (T6); Group (Y + B) = 0.4Y + 0.4B (T7), 0.4Y + 0.5B (T8), 0.4Y + 0.6B (T9), 0.5Y + 0.4B (T10), 0.5Y + 0.5B (T11), 0.5Y + 0.6B (T12), 0.6Y + 0.4B (T13), 0.6Y + 0.5B (T14), 0.6Y + 0.6B (T15). Yogurt cultures (Y) (*Streptococcus thermophilus* and *Lactobacillus bulgaricus*) and bifidobacterium cultures (B) (*Bifidobacterium bifidum* and *Bifidobacterium longum*)

Colonies of bifidobacteria (BC) were enumerated in LFC as shown in Figure 4. Control and group Y did not show any bifidobacteria colonies, because they were not added to those treatments during cheese manufacture. For the other groups, total BC gradually increased up to 15 d, following by a decrease till the 30 d of storage. The total BC in group B was higher than other treatments, where T5 had the highest BC while T15 had the lowest BC. The BC in T5 increased from 6.32 log cfu/g to 7.43 log cfu/g after 15 d of storage, and then, the number decreased to 6.83 log cfu/g by the end of the storage. The BC in T15 increased from 5.15 to 6.46 log cfu/g after 15 d of storage and then decreased to 5.88 log cfu/g after 30 d. The gradual decrease in viable bifidobacteria after 15 d of storage may be due to the effect of higher acidity and the presence of lactic and acetic acids that may have affected the viability of BC during the ripening period (Dave & Shah, 1998). The obtained results are in agreement with other studies where researchers had found that numbers of bifidobacterium declined slowly after 21 d, followed by a sharp decline in their numbers toward the end of the ripening of soft cheese (Gomes & Malcata, 1998). The decline in BC at the end of the storage time could be resulted from the low resistance of bifidobacteria with increasing the acidity values after 30 d, as we reported in a previous study (Ahmed et al., 2020). However, the BC ranged from approximately 5 to 7 log cfu/g, which is required to consider the LFC as a probiotic product.

**FIGURE 5:**
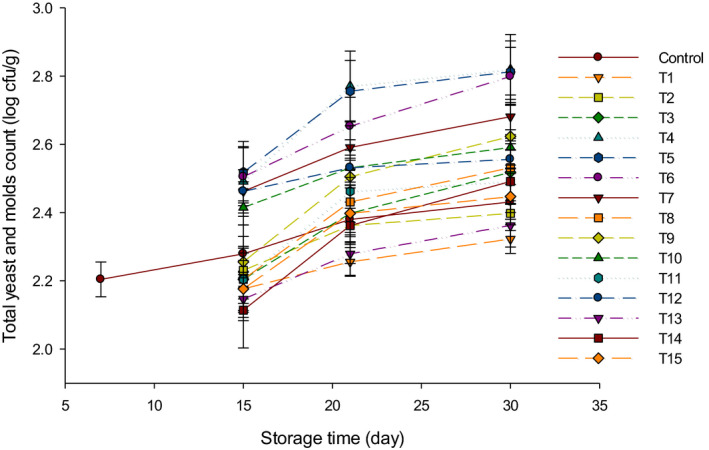
The total yeast and molds counts (log cfu/g) of low‐fat Feta cheese (LFC) during 30 d of storage period. Control = Cheese made with only rennet; Group (Y) = 0.4% (T1), 0.5% (T2), and 0.6% (T3); Group (B) = 0.4% (T4), 0.5% (T5), and 0.6% (T6); Group (Y + B) = 0.4Y + 0.4B (T7), 0.4Y + 0.5B (T8), 0.4Y + 0.6B (T9), 0.5Y + 0.4B (T10), 0.5Y + 0.5B (T11), 0.5Y + 0.6B (T12), 0.6Y + 0.4B (T13), 0.6Y + 0.5B (T14), 0.6Y + 0.6B (T15). Yogurt cultures (Y) (*Streptococcus thermophilus* and *Lactobacillus bulgaricus*) and bifidobacterium cultures (B) (*Bifidobacterium bifidum* and *Bifidobacterium longum*)

Yeast and mold count (log cfu/g) of probiotic LFC is presented in Figure 5. The yeast and mold count in control was detected after 7 d of storage period. Nevertheless, the yeast and molds in other treatments were detected in lower numbers after 15 d of storage at 4°C. A few colonies of fungi or surface microorganisms have appeared after 21 d of storage in some samples, especially in group B cheeses. This might be caused by the commitment to hygienic practices during the manufacturing of cheese. The number of yeast and mold initiated to increase by extending the storage period as a result of acidity development (Makhal, Kanawjia, & Giri, 2015). Generally, these microorganisms are proliferated in the manufacturing environment, which can allow them to reach the cheese samples during manufacture (Vinderola, Prosello, Ghiberto, & Reinheimer, 2000).

Tests were carried out to estimate the coliform group in all LFC treatments. The results were negative in all those tests for all treatments during the 30 d of storage period. This is due to the pasteurization of milk before the manufacturing of cheese, which eliminates coliform bacteria. Moreover, the hygienic practices during the experimental procedure of probiotic LFC for all treatments inhibit the growth of coliforms. These results are in agreement with the finding of several investigators (Ordóñez, Ibáñez, Torre, & Barcina, 1999).

### 3.2 Sensory evaluation

#### 3.2.1 Color and appearance

**FIGURE 6:**
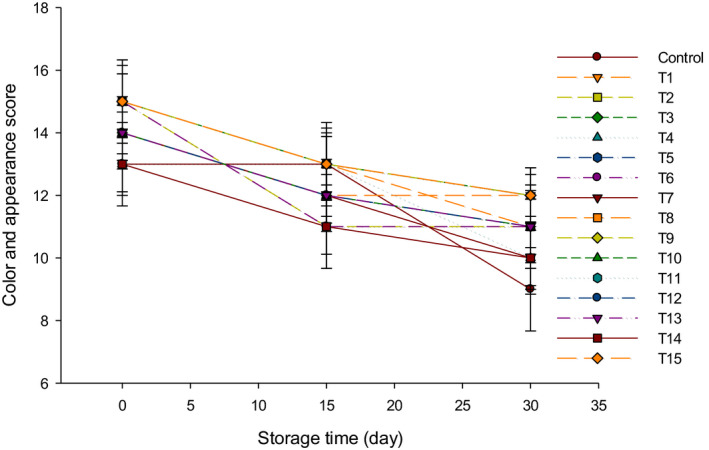
The color and appearance scores of low‐fat Feta cheese (LFC) during 30 d of storage period. Control = Cheese made with only rennet; Group (Y) = 0.4% (T1), 0.5% (T2), and 0.6% (T3); Group (B) = 0.4% (T4), 0.5% (T5), and 0.6% (T6); Group (Y + B) = 0.4Y + 0.4B (T7), 0.4Y + 0.5B (T8), 0.4Y + 0.6B (T9), 0.5Y + 0.4B (T10), 0.5Y + 0.5B (T11), 0.5Y + 0.6B (T12), 0.6Y + 0.4B (T13), 0.6Y + 0.5B (T14), 0.6Y + 0.6B (T15). Yogurt cultures (Y) (*Streptococcus thermophilus* and *Lactobacillus bulgaricus*) and bifidobacterium cultures (B) (*Bifidobacterium bifidum* and *Bifidobacterium longum*)

The color and appearance scores of LFC with different starter cultures are presented in Figure 6. The color and appearance of all cheeses did not markedly affect (*p* > .05) by adding starter cultures since the cheese was kept at 4°C. These results were in agreement with other studies, which reported that using *Bifidobacterium bifidum* in Cheddar cheese manufacturing did not increase the metabolic action and did not affect the cheese color during the 24 weeks of ripening (Dinakar & Mistry, 1994).

#### 3.2.2 Body and texture

**FIGURE 7:**
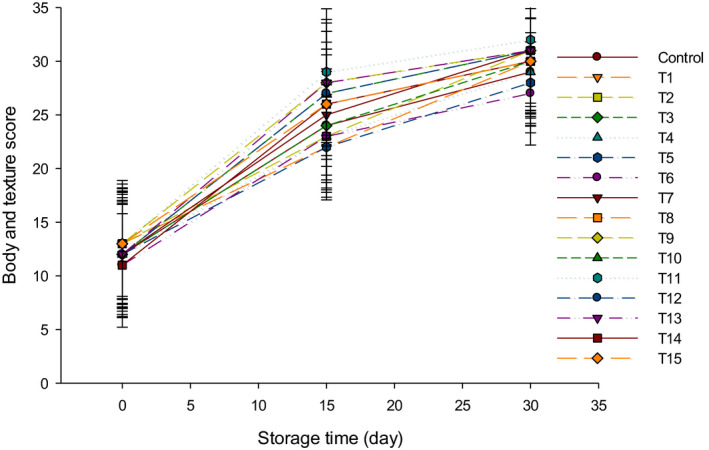
The body and texture scores of low‐fat Feta cheese (LFC) during 30 d of storage period. Control = Cheese made with only rennet; Group (Y) = 0.4% (T1), 0.5% (T2), and 0.6% (T3); Group (B) = 0.4% (T4), 0.5% (T5), and 0.6% (T6); Group (Y + B) = 0.4Y + 0.4B (T7), 0.4Y + 0.5B (T8), 0.4Y + 0.6B (T9), 0.5Y + 0.4B (T10), 0.5Y + 0.5B (T11), 0.5Y + 0.6B (T12), 0.6Y + 0.4B (T13), 0.6Y + 0.5B (T14), 0.6Y + 0.6B (T15). Yogurt cultures (Y) (*Streptococcus thermophilus* and *Lactobacillus bulgaricus*) and bcultures (B) (*Bifidobacterium bifidum* and *Bifidobacterium longum*)

The body and texture scores of LFC with different starter cultures are presented in Figure 7. The starter cultures had a slight effect on the body and texture of LFC which gained higher scores during the ripening time. During storage, the hard casein matrix turned to soft texture because of the slow proteolysis under the action of the endogenous enzymes, rennet enzymes, and also from the added starters proteolytic enzymes. The LFC made with mixed groups (Y + B) gained the highest body and texture scores as compared to other groups (Figure 7), which may be due to the low moisture content in that group (Ahmed et al., 2020). The body and texture of group B were comparatively softer than group Y and group Y + B. This could be due to the low acidity levels in group B that led to retain the high moisture content during the 30 d of storage (Ahmed et al., 2020). It has been reported that using mixed cultures of *Streptococcus thermophilus* and Bifidobacterium improved the body and texture of Damiata cheese (Feta cheese type) during the storage period (Boylston, Vinderola, Ghoddusi, & Reinheimer, 2004).

#### 3.2.3 Flavor

**FIGURE 8:**
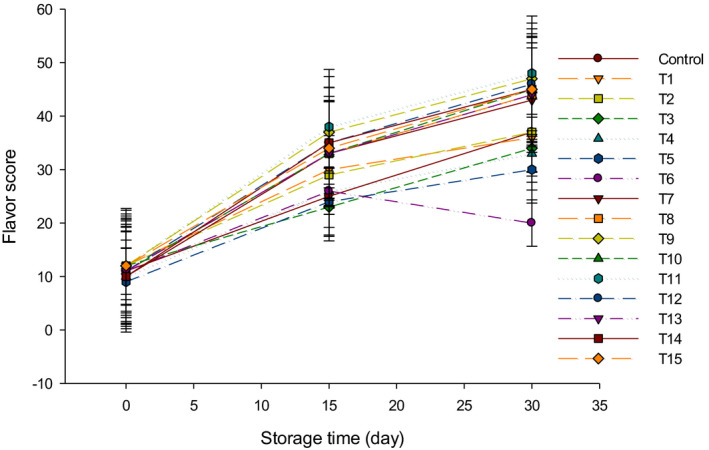
The flavor scores of low‐fat Feta cheese (LFC) during 30 d of storage period. Control = Cheese made with only rennet; Group (Y) = 0.4% (T1), 0.5% (T2), and 0.6% (T3); Group (B) = 0.4% (T4), 0.5% (T5), and 0.6% (T6); Group (Y + B) = 0.4Y + 0.4B (T7), 0.4Y + 0.5B (T8), 0.4Y + 0.6B (T9), 0.5Y + 0.4B (T10), 0.5Y + 0.5B (T11), 0.5Y + 0.6B (T12), 0.6Y + 0.4B (T13), 0.6Y + 0.5B (T14), 0.6Y + 0.6B (T15). Yogurt cultures (Y) (*Streptococcus thermophilus* and *Lactobacillus bulgaricus*) and bifidobacterium cultures (B) (*Bifidobacterium bifidum* and *Bifidobacterium longum*)

**FIGURE 9:**
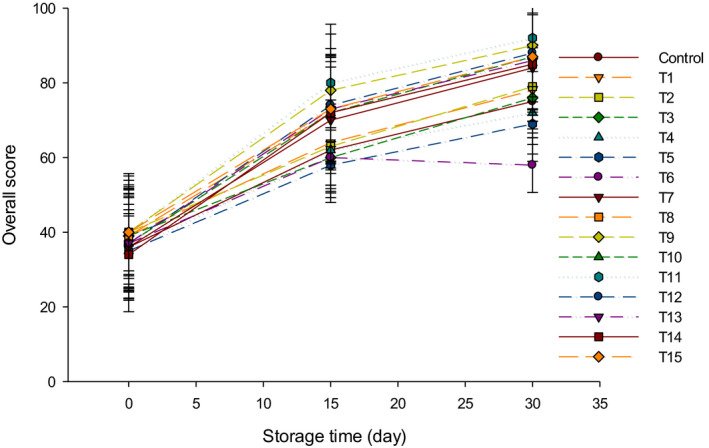
The overall scores of low‐fat Feta cheese (LFC) during 30 d of storage period. Control = Cheese made with only rennet; Group (Y) = 0.4% (T1), 0.5% (T2), and 0.6% (T3); Group (B) = 0.4% (T4), 0.5% (T5), and 0.6% (T6); Group (Y + B) = 0.4Y + 0.4B (T7), 0.4Y + 0.5B (T8), 0.4Y + 0.6B (T9), 0.5Y + 0.4B (T10), 0.5Y + 0.5B (T11), 0.5Y + 0.6B (T12), 0.6Y + 0.4B (T13), 0.6Y + 0.5B (T14), 0.6Y + 0.6B (T15). Yogurt cultures (Y) (*Streptococcus thermophilus* and *Lactobacillus bulgaricus*) and bifidobacterium cultures (B) (*Bifidobacterium bifidum* and *Bifidobacterium longum*)

The flavor scores of LFC made with different types of starter cultures are shown in Figure 8. The flavor of all cheeses was improved over 30 d of storage at 4°C. Generally, the mixed starter culture cheeses (Y + B) gained the highest flavor and aroma scores since the combination of both bacteria improved the overall flavor than group Y and group B cheeses. Group Y gained higher flavor scores as compared to group B (Figure 8). This may be due to the high metabolic activities of yogurt starter cultures that may produce flavor compounds similar to those in fermented milk. For this reason, the trained panelists gave higher scores to LFC made with group Y as compared to group B. These results were in agreement with those obtained by Hammam, Tammam, and El‐Rahim (2018), who reported that the flavor of Ras cheese made using *Streptococcus thermophilus* and *Lactobacillus bulgaricus* improved during the ripening period. Group B cheeses had gained the lowest flavor and aroma scores since bifidobacteria do not produce flavor components but acetate (Ong, Henriksson, & Shah, 2007). It is also known that bifidobacteria are poorly grown in milk and its products (Peirotén et al., 2019). The panelists tasted the acetic acid flavor, which proportionally increased as the percentage of bifidobacteria starter cultures elevated (Ong et al., 2007). The panelists did not taste the acetic acid flavor in the cheese produced from a mix of Y + B which resulted in cheese with a higher flavor score as compared to group B that had lowest flavor scores. It has been reported that the flavor of Festivo low‐fat cheese improved using bifidobacteria and *Streptococcus thermophilus* (Ryhänen, Pihlanto‐Leppälä, & Pahkala, 2001). It was expected that rennet and indigenous enzymes had an important role to produce the flavor in LFC during cheese ripening (Pereira, Gomes, Gomes, & Malcata, 2008). The overall acceptability of LFC is shown in Figure 9. The highest scores (92/100) were found in T11 after 30 d of storage, followed by T9 (90/100), T12 (88/100), and lastly for the rest of cheeses produced with group Y + B starter cultures.

## 4 CONCLUSION

The LFC was manufactured using Y, B, and Y + B cultures at rates of 0.4, 0.5, and 0.6%, respectively. We concluded that a mix of Y + B cultures improved the flavor and texture of LFC, especially at 0.4 + 0.6% and 0.5 + 0.5% proportions which can be used to produce LFC with a flavor profile similar to FFC. Additionally, the Y + B starter cultures resulted in LFC with higher lactic acid bacteria up to 30 d of storage. Probiotic bacteria ranged from 5 to 7 log cfu/g in the LFC, so it can provide potential health benefits to consumers. This study indicates that the LFC can be produced with a typical FFC flavor profile by selecting an appropriate ratio of Y + B starter cultures.

## CONFLICT OF INTEREST

The authors have no conflict of interest to declare.
